# Supplementary material for: Epithelial plasticity can generate multi-lineage phenotypes in human and murine bladder cancers
Source: Nat Commun. 2020 May 21;11:2540. doi: 10.1038/s41467-020-16162-3 (PMC7242345; doi:10.1038/s41467-020-16162-3)
Supplement: Supplementary file 1 — Supplementary Information [file 41467_2020_16162_MOESM1_ESM.pdf]

Supplementary Information. Sfakianos *et al*., 2020

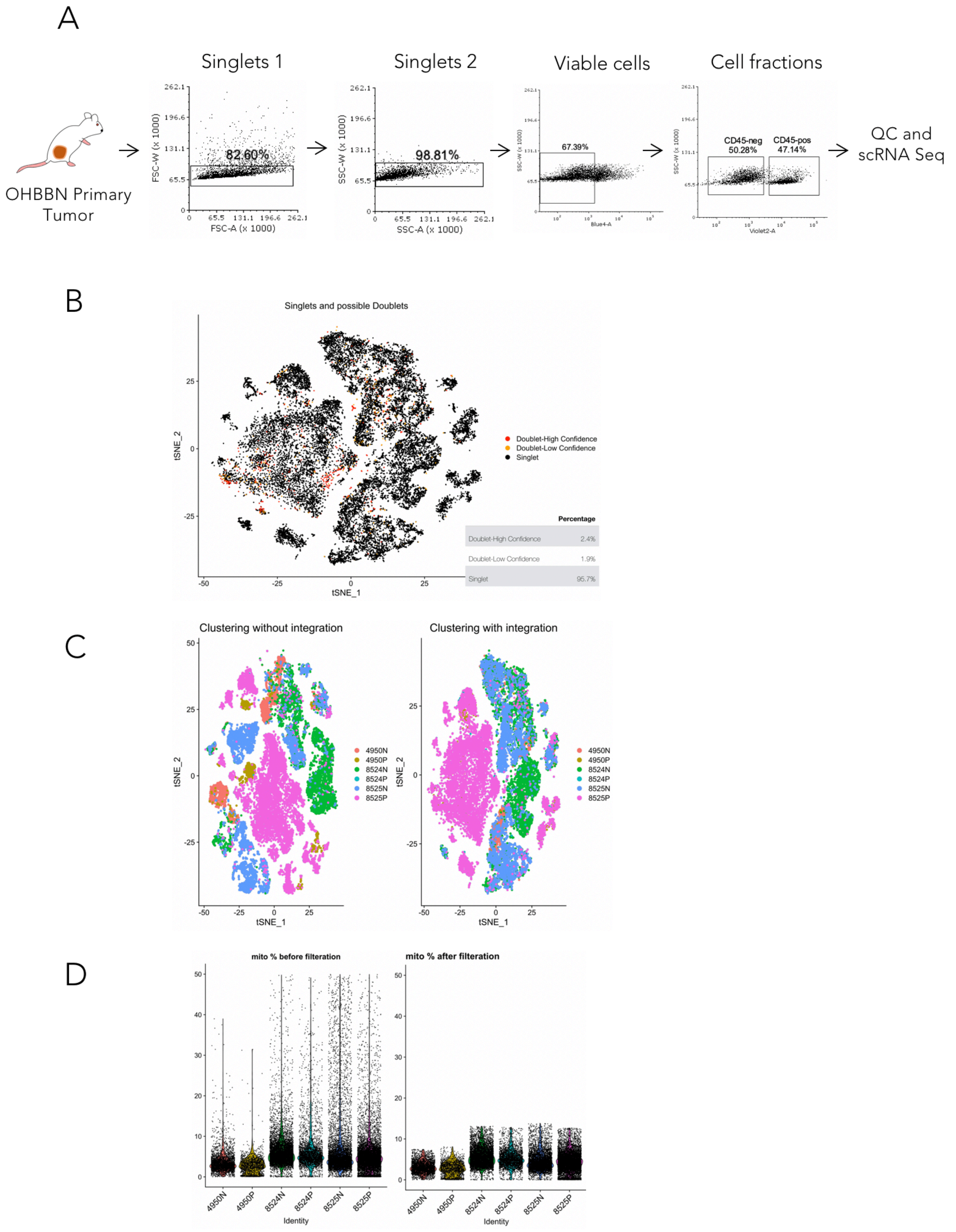

Supplementary Figure 1. Quality control for single cell RNA Sequencing analysis. **A,** Strategy for isolating CD45-negative and CD45-positive cells in mouse tumors for scRNA-seq analysis. **B,** Detection of potential doublets using Doublet Finder Tool showing less than 5% possible doublets existing outside of epithelial clusters. **C**, tSNE plots showing clusters without and with integration. **D**, Percentage of mitochondrial DNA before and after filtration.

A. Mouse

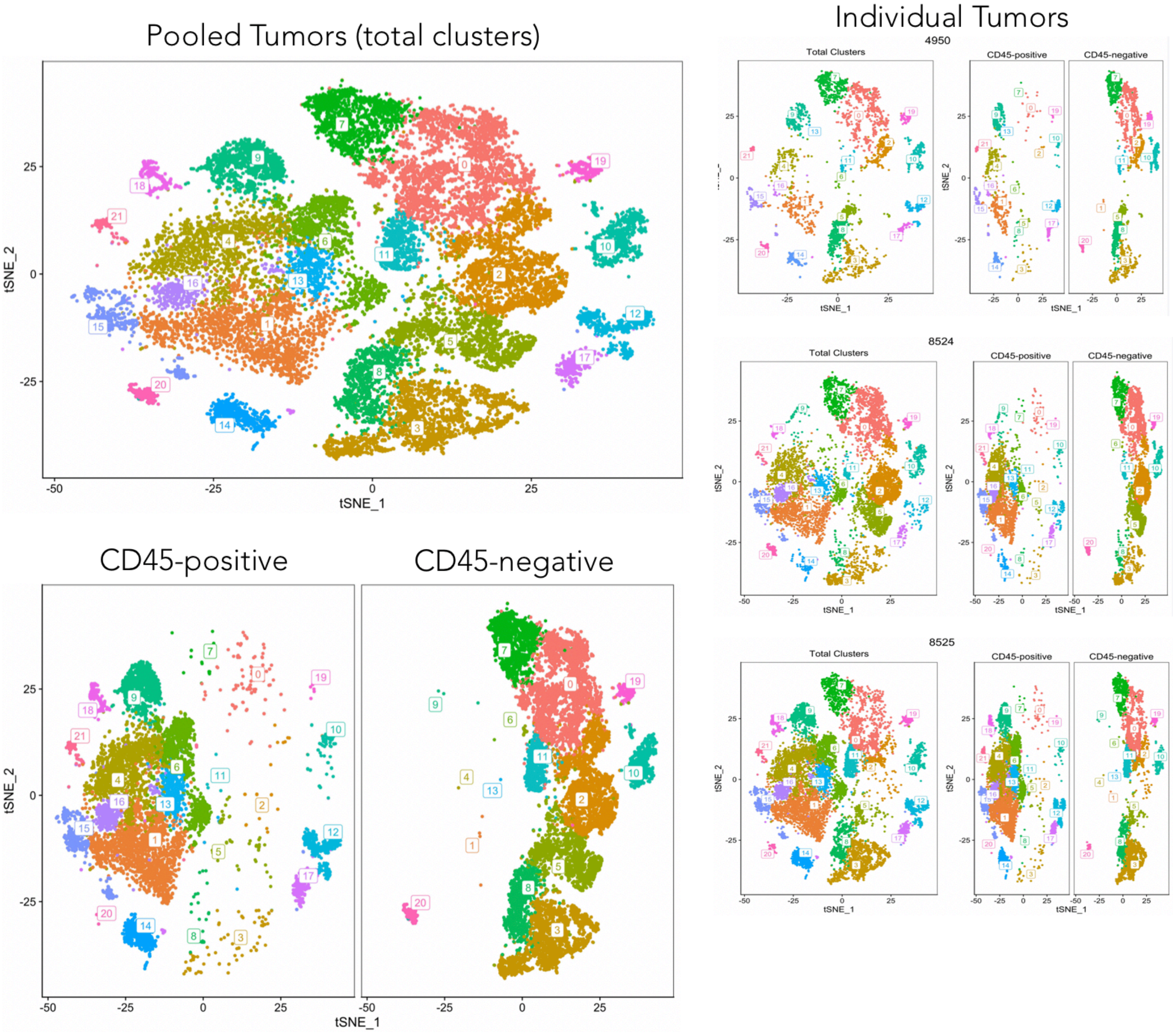

B. Human

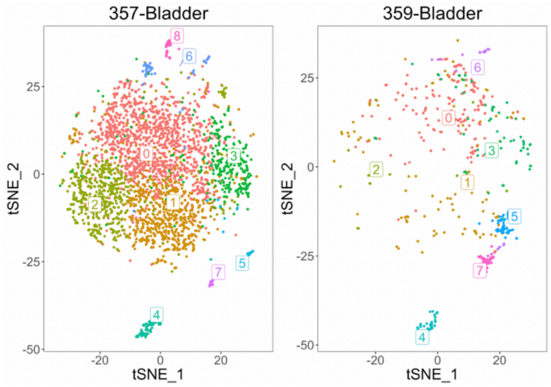

Supplementary Figure 2. Clustering analysis for mouse and human bladder tumors. **A,** tSNE plots for pooled OHBBN mouse tumors shown as total clusters or as separated CD45-negative and CD45-positive fractions (left). tSNE plots for individual mouse tumors are shown on the right. A total of 22 clusters were identified. **B,** tSNE plots are shown for individual human tumors.

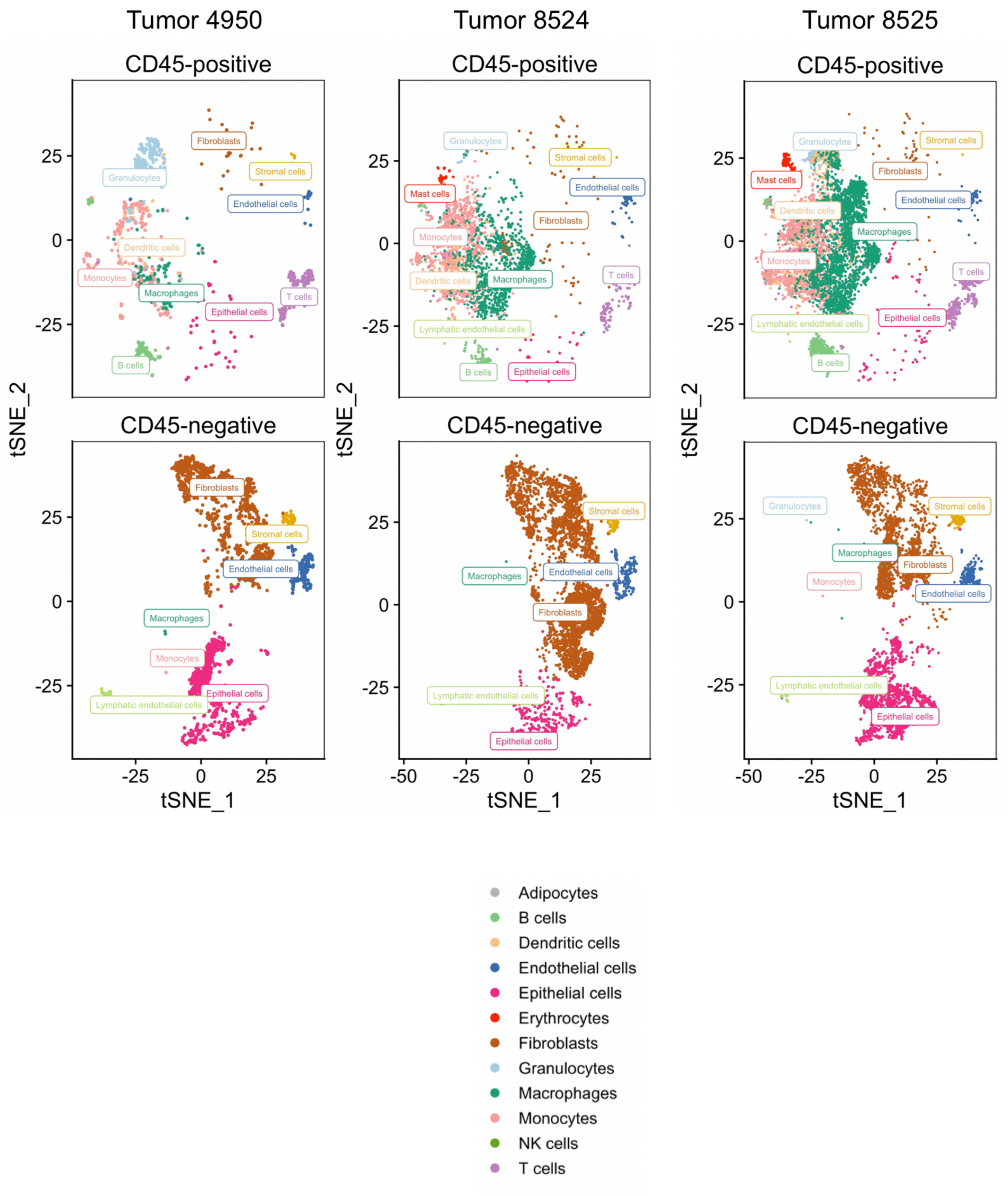

Supplementary Figure 3. tSNE plots showing cell types for analyzed primary mouse tumors (n=3) separated into CD45-positive and CD45-negative cells.

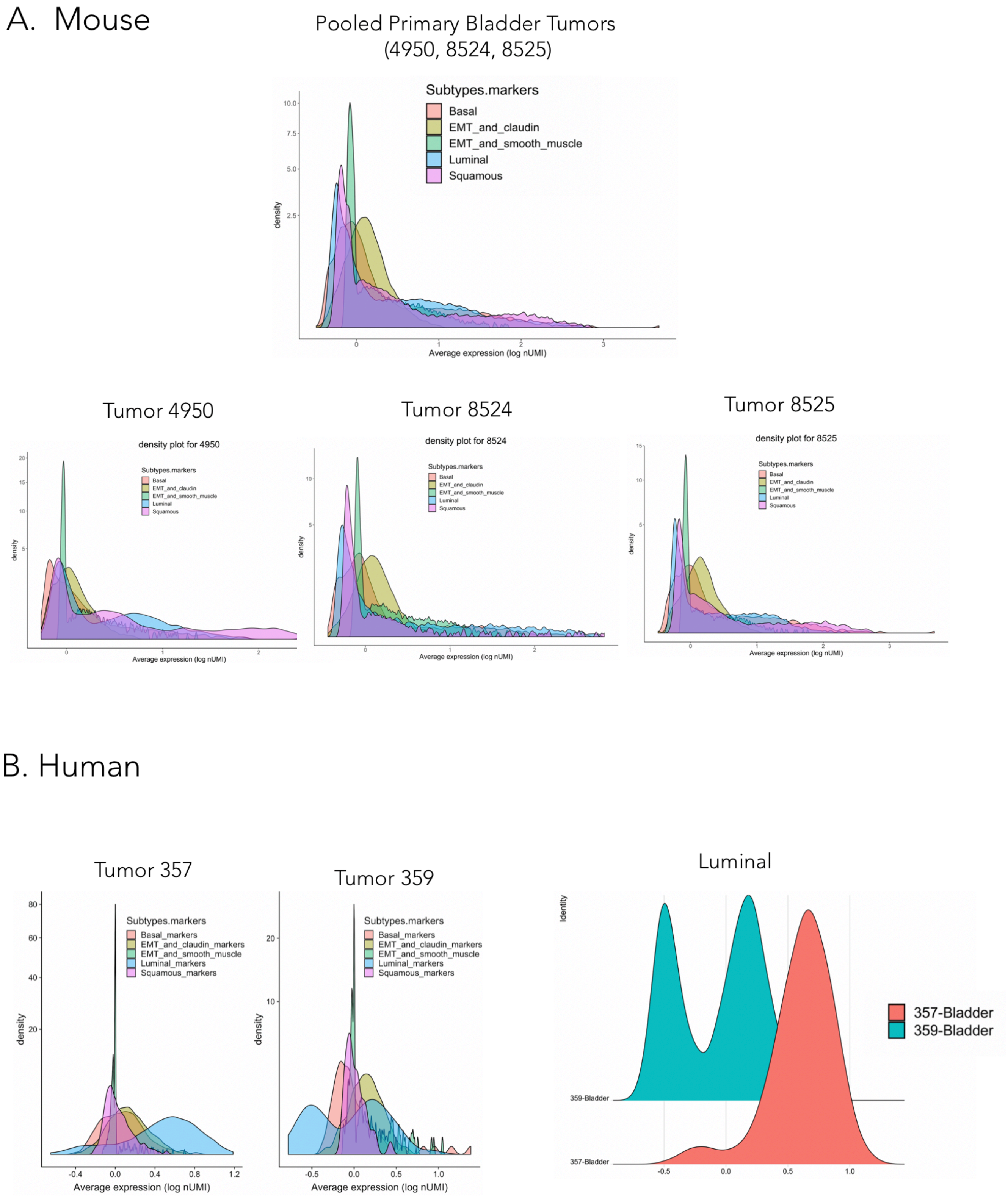

Supplementary Figure 4. Lineage density graphs showing expression of marker genes for mouse and human bladder tumors. **A,** Pooled OHBBN tumors and density graphs as a function of average gene expression for five lineage subtypes (top) and individual tumors (lower). **B,** Lineage density graphs for two individual human bladder tumors (left) and individual analysis confirming more luminal gene expression in tumor 359 compared to tumor 357.

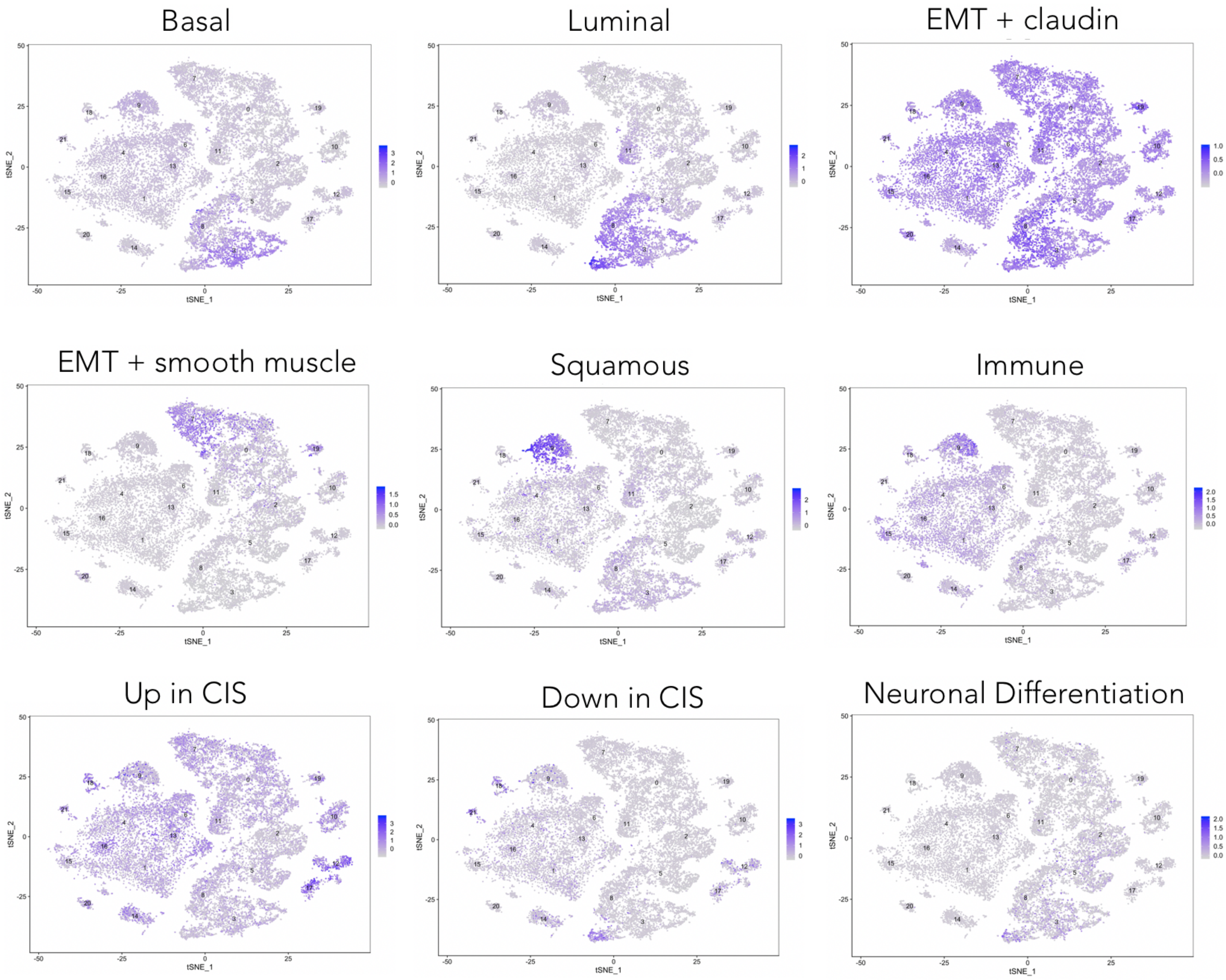

Supplementary Figure 5. Single pathway analysis in pooled primary bladder tumors shown in tSNE plots. Genes used to generate tSNE plots include: basal *(Cd44, Cdh3, Krt1, Krt14, Krt16, Krt5, Krt6a, Krt6b, Krt6c)*, luminal *(Cyp2j2, Erbb2, Erbb3, Fgfr3, Foxa1, Gata3, Gpx2, Krt18, Krt19, Krt20, Krt7, Krt8, Pparg, Xbp1, Upk1a, Upk2)*, EMT + claudin *(Zeb1, Zeb2, Vim, Snai1, Snai2, Twist1, Foxc2, Cdh1, Cdh2, Cldn3, Cldn4, Cldn7)*, EMT + smooth muscle *(Pgm5, Des, C7, Sfrp4, Comp, Sgcd)*, squamous *(Dsc1, Dsc2, Dsc3, Dsg1, Dsg2, Dsg3, S100a7, S100a8)*, immune *(Cd274, Pdcd1lg2, Ido1, Cxcl11, L1cam, Saa1)*, up in CIS *(Msn, Nr3c1)*, down in CIS *(Crtac1, Ctse, Padi3)* and neuronal differentiation *(Msi1, Plekhg4b, Gng4, Peg10, Rnd2, Aplp1, Sox2, Tubb2b).*

A Pooled Primary Bladder Tumors (4950, 8524, 8525)

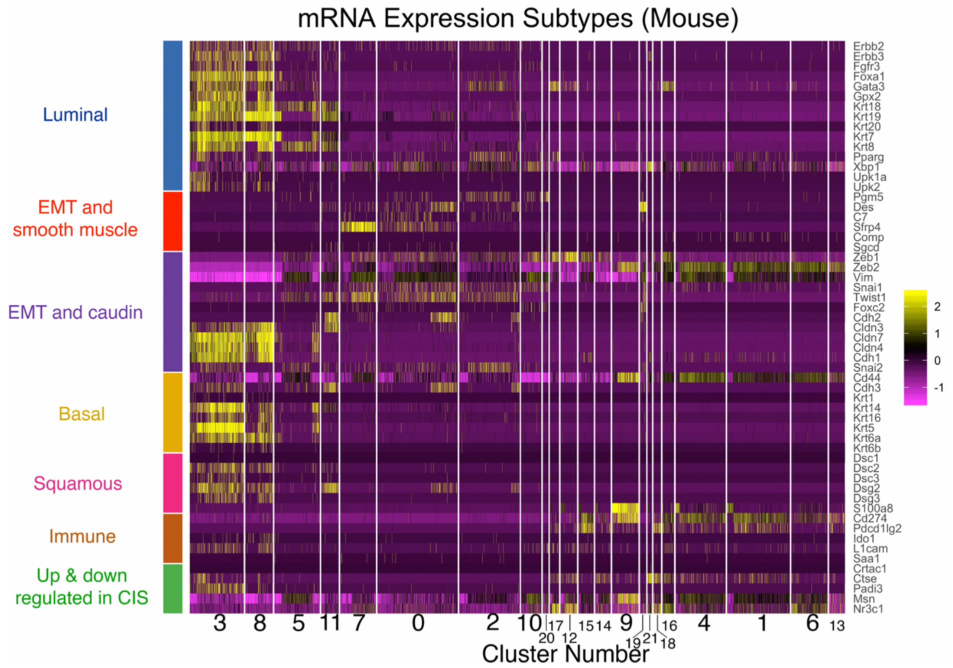

B

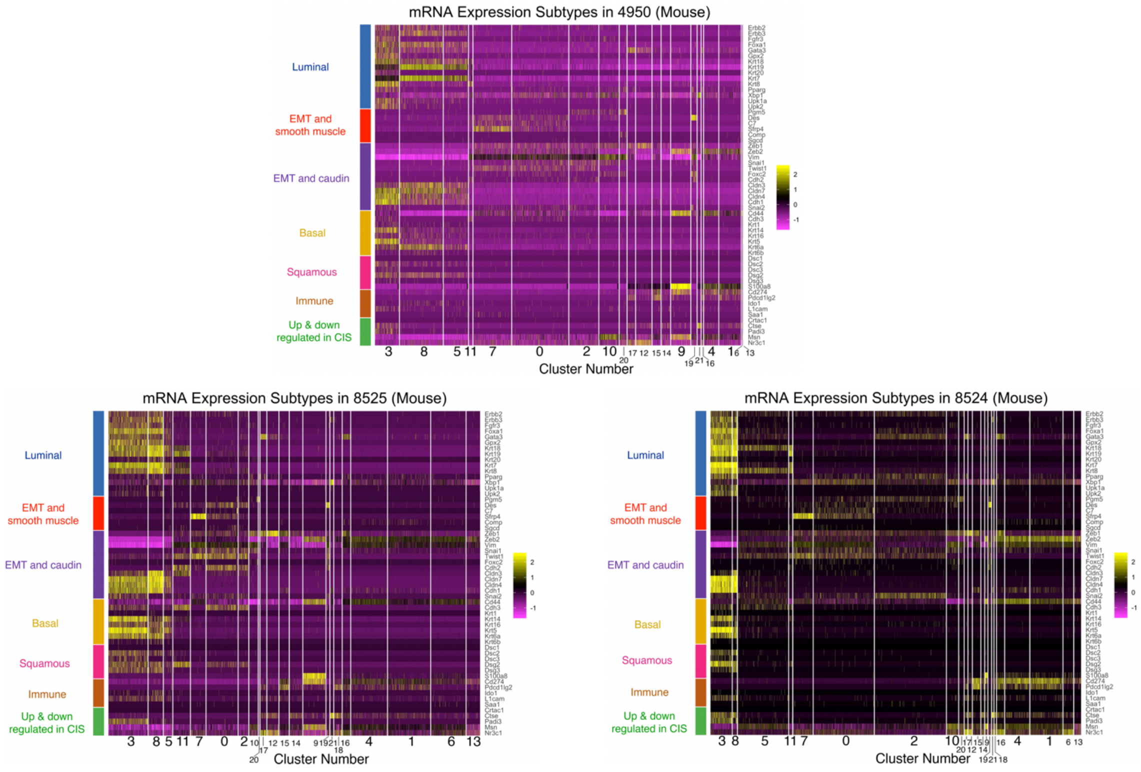

Supplementary Figure 6. Heat maps showing lineage marker mRNA expression subtypes (vertical axis) as a function of tSNE clusters (horizontal axis) analyzed in, **A**, pooled primary and, **B**, individual OHBBN induced mouse bladder tumors.

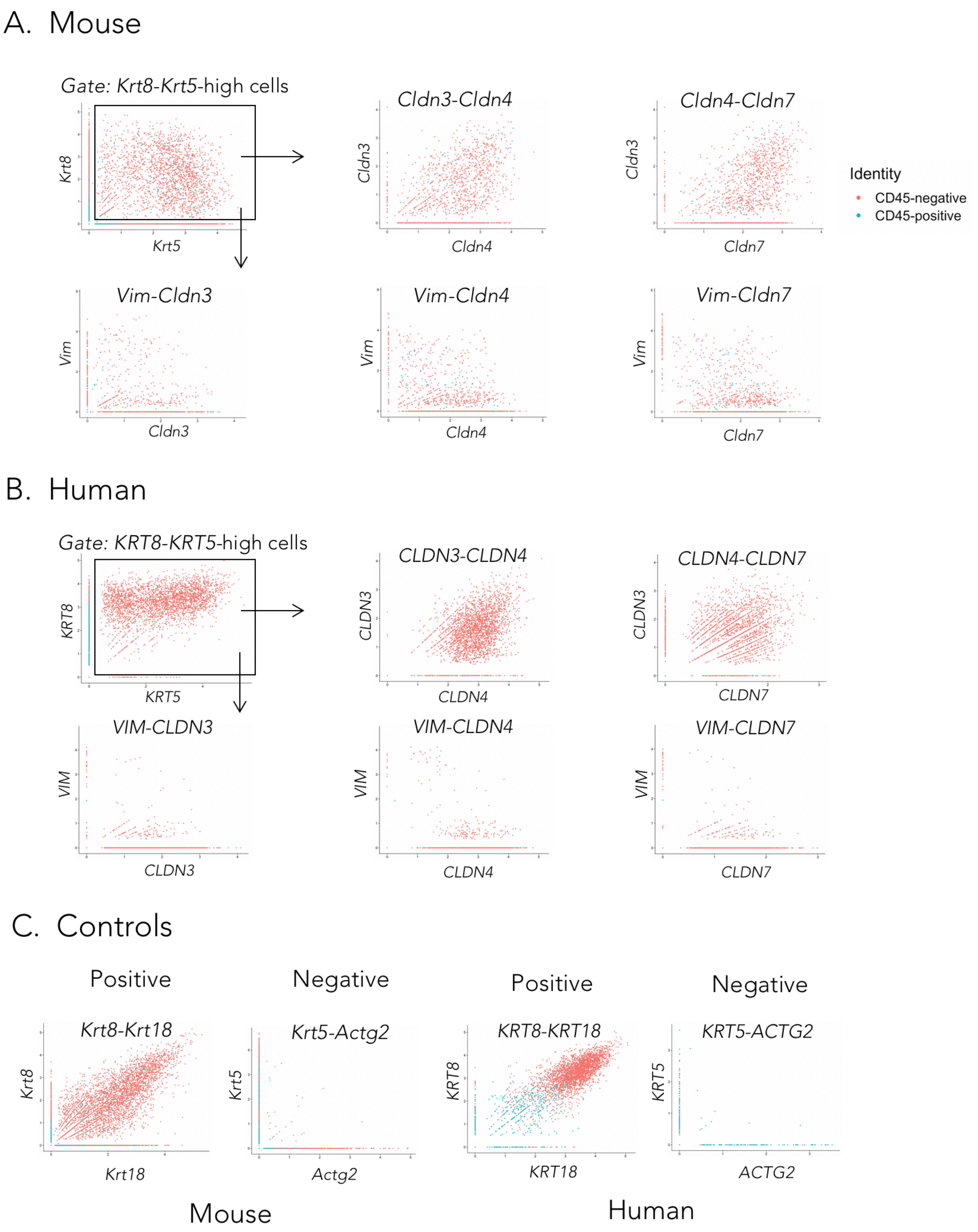

Supplementary Figure 7. Gene plots showing that *Krt5-Krt8* high cells are high in EMT-claudin gene expression. Single cell gene plots gated on cells with *Krt5-Krt8* gene expression followed by assessment for positive expression of EMT-claudin genes (*Cldn4*, *Cldn7*, *Vim*) in, **A**, mouse and, **B**, human bladder tumors. **C**, Positive (*Krt8-Krt18*) and negative (*Krt5-Actg2*) control gene plots for mouse and human analysis. Cells with Krt5 >0 and Krt8>0 were gated as Krt5-Krt8 high. Cells with Krt5 = 0 and Krt8 = 0 were gated as Krt5-Krt8 low. Axis units are log (UMI) or transformed transcripts per cell.

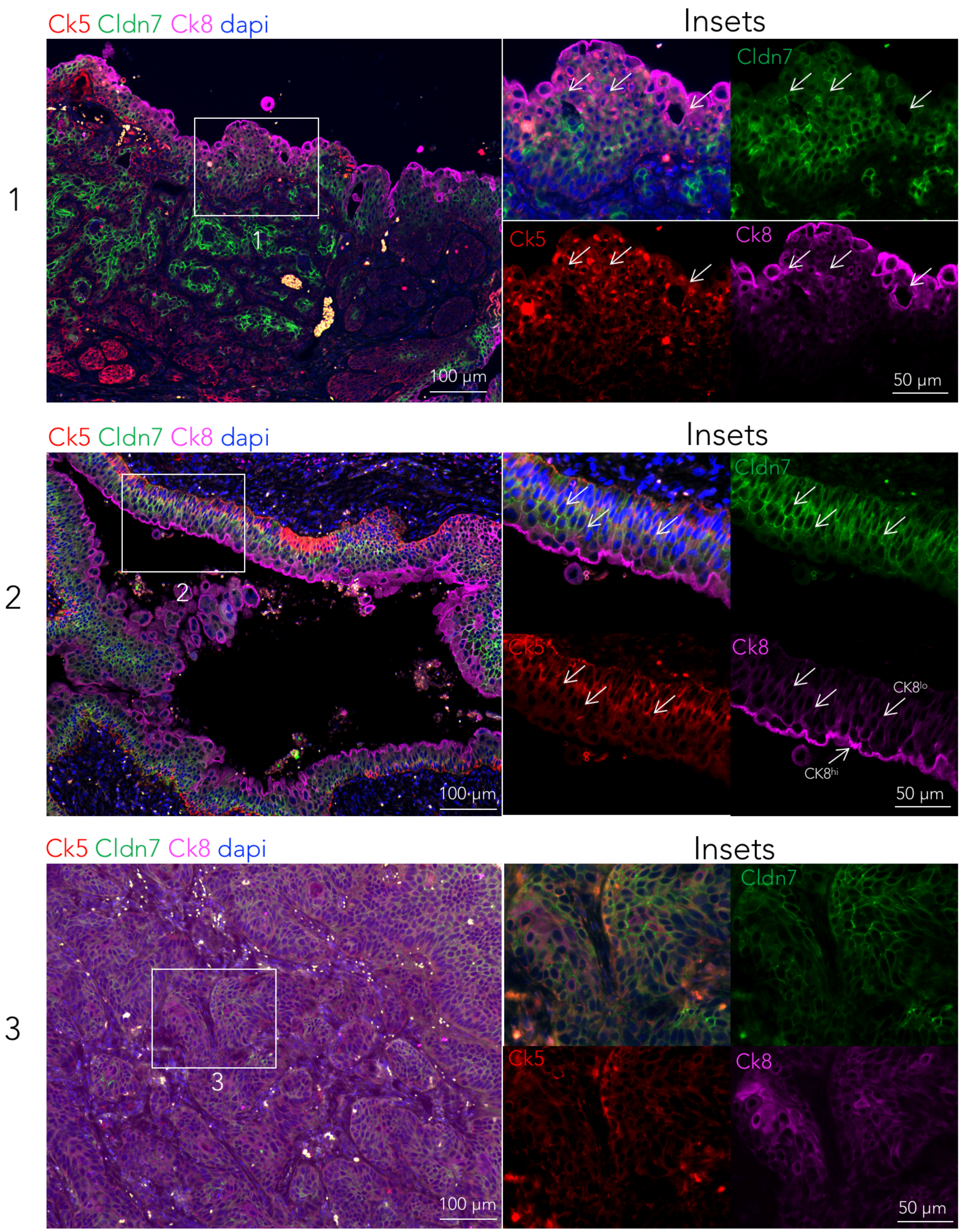

Supplementary Figure 8. Detection of OHBBN induced mouse bladder cancer cells with single, double and triple lineage marker positive cells. Representative triple labelling immunofluorescence showing the co-distribution of basal: CK5, p40 (Alexa 546); luminal: CK8 (Alexa 647) and EMT-claudin Cldn7 (Alexa 546) in panels 1-4. Panel 6 shows the same antibodies but using CK5 (Alexa 488), CK8 (Alexa 546) and Cldn7 (Alexa 647). Immunostaining experiments were conducted on six different mouse tumors.

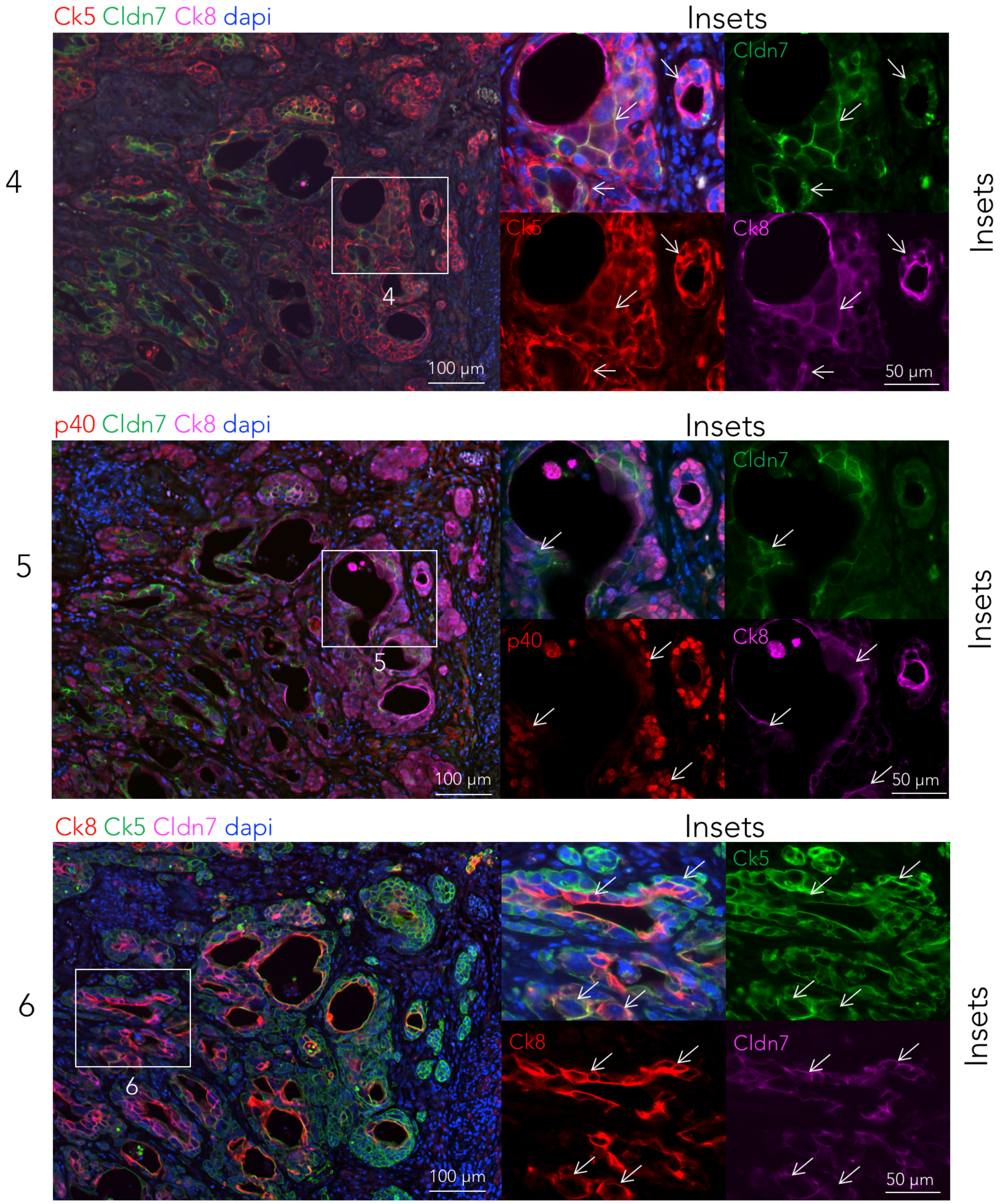
Ck5 Cldn7 Ck8 dapi
Insets
4
Cldn7
Ck5
Ck8
100 µm
50 µm
Insets
p40 Cldn7 Ck8 dapi
Insets
5
Cldn7
p40
Ck8
100 µm
50 µm
Insets
Ck8 Ck5 Cldn7 dapi
Insets
6
Ck5
Ck8
Cldn7
100 µm
50 µm
Insets

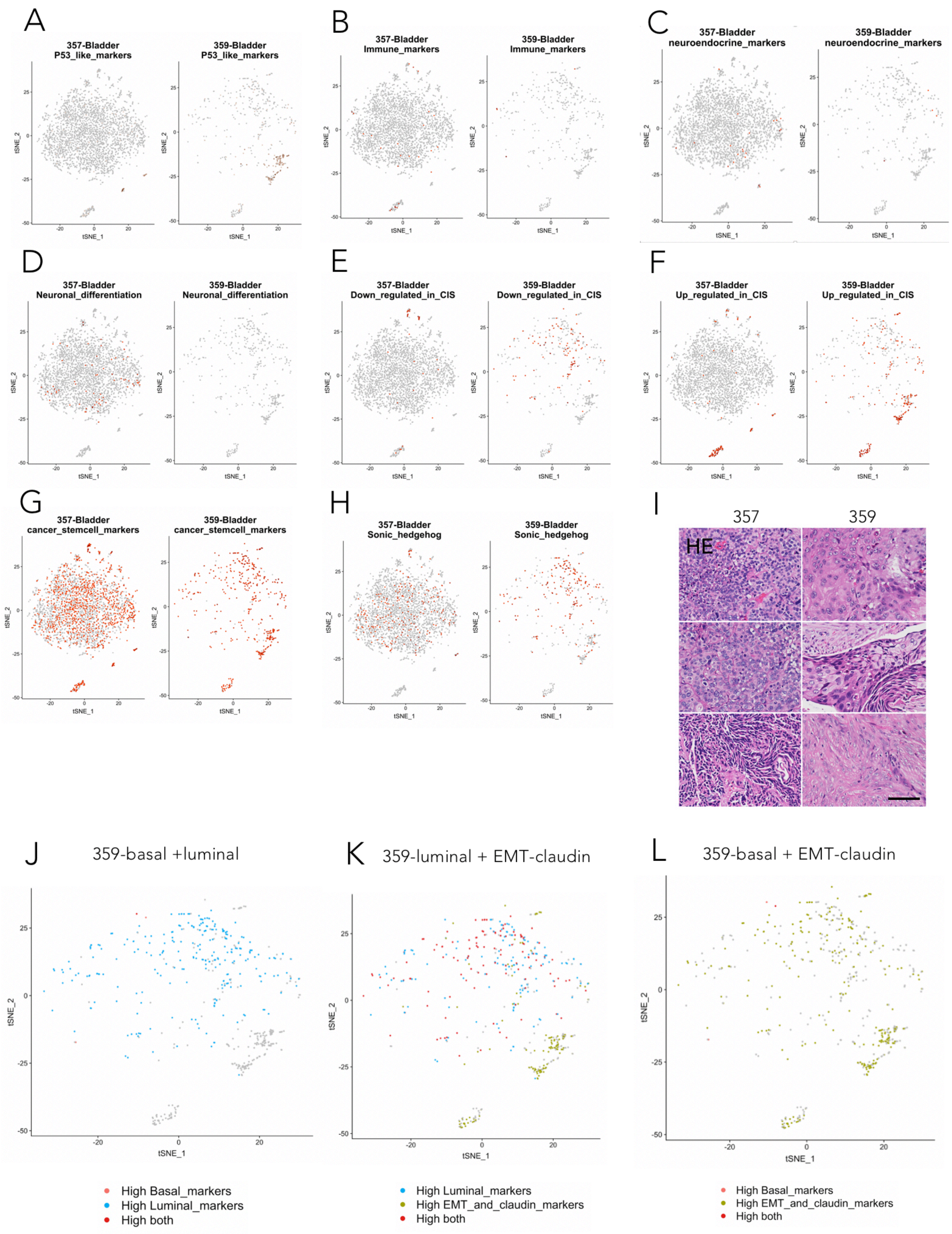

Supplementary Figure 9. tSNE plots of human bladder tumors showing gene expressions for marker panels including: **A,** P53-like, **B**, immune, **C**, neuroendocrine, **D**, neuronal differentiation, **E**, down regulated in CIS, **F**, upregulated in CIS, **G**, cancer stem cell and, **H**, sonic hedgehog signaling, **I**, HE images from human tumor samples 357 and 359 (bar = 100 μM). **J-L**, tSNE plots in tumor 359 showing the presence of luminal + EMT-claudin, bi-lineage positive cells. Human pathologies were assessed from two independent human tumors.

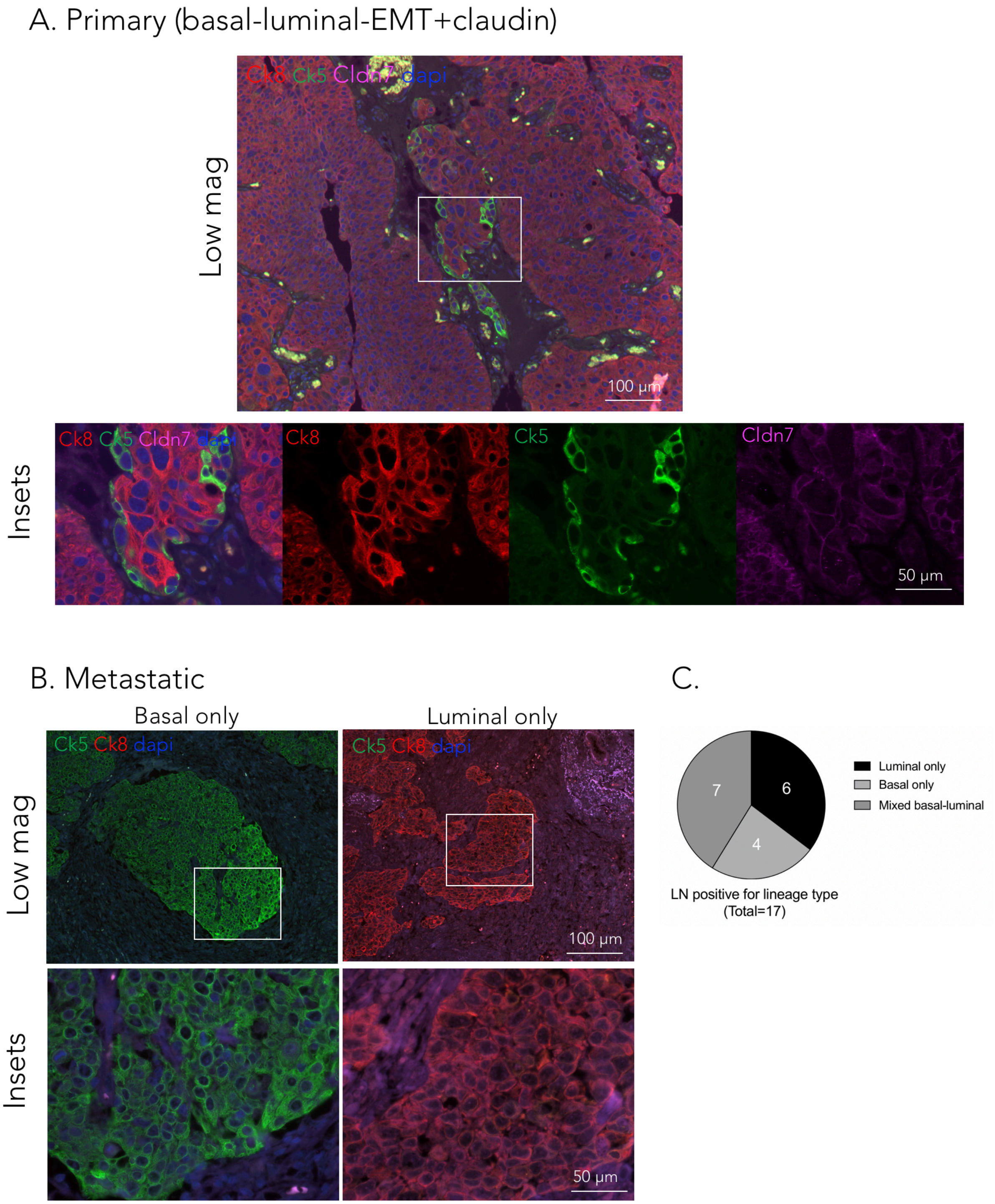

Supplementary Figure 10. Human primary and metastatic bladder cancer samples can be single or mixed lineage positive. **A,** Primary bladder tumors showing the coexistence of basal, luminal and EMT-claudin marker positive cells. **B,** Representative images of LN metastasis with high basal (low luminal) and high luminal (low basal) cells shown at low and high magnification. **C,** Frequency of detection for single lineage versus mixed lineage metastatic bladder cancer in the lymph node (LN) (n=17). Examples of mixed lineage LN metastasis having, **D**, basal and luminal positive cells and, **E**, basal, luminal and EMT-claudin positive cells shown at low and high magnification. At least 15 primary human primary bladder and 17 metastatic bladder tumors were assessed.

D. Metastatic (basal-luminal)

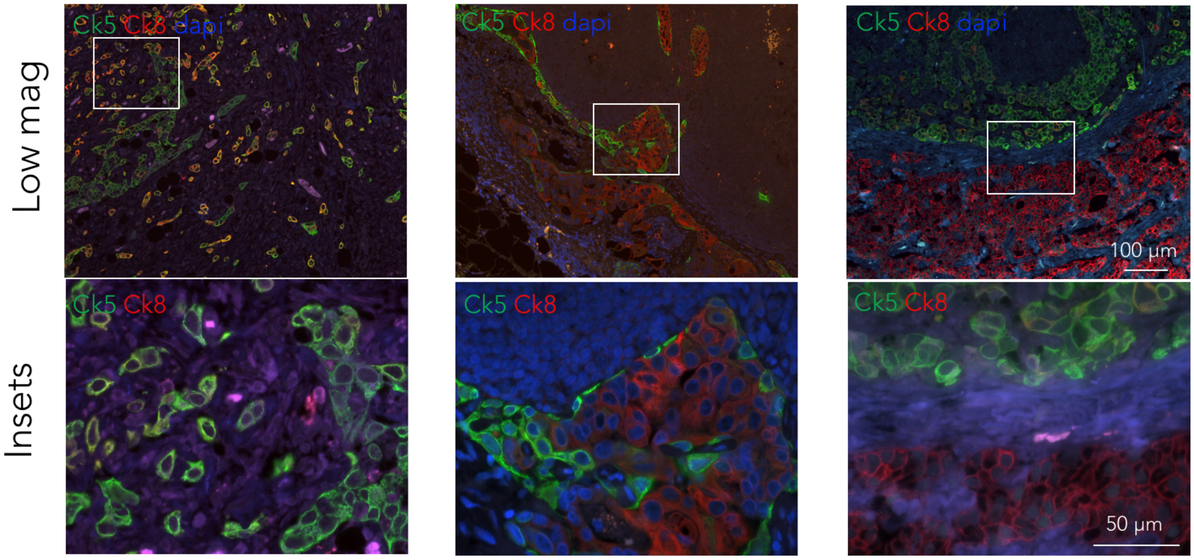

E. Metastatic (basal-luminal-EMT+claudin)

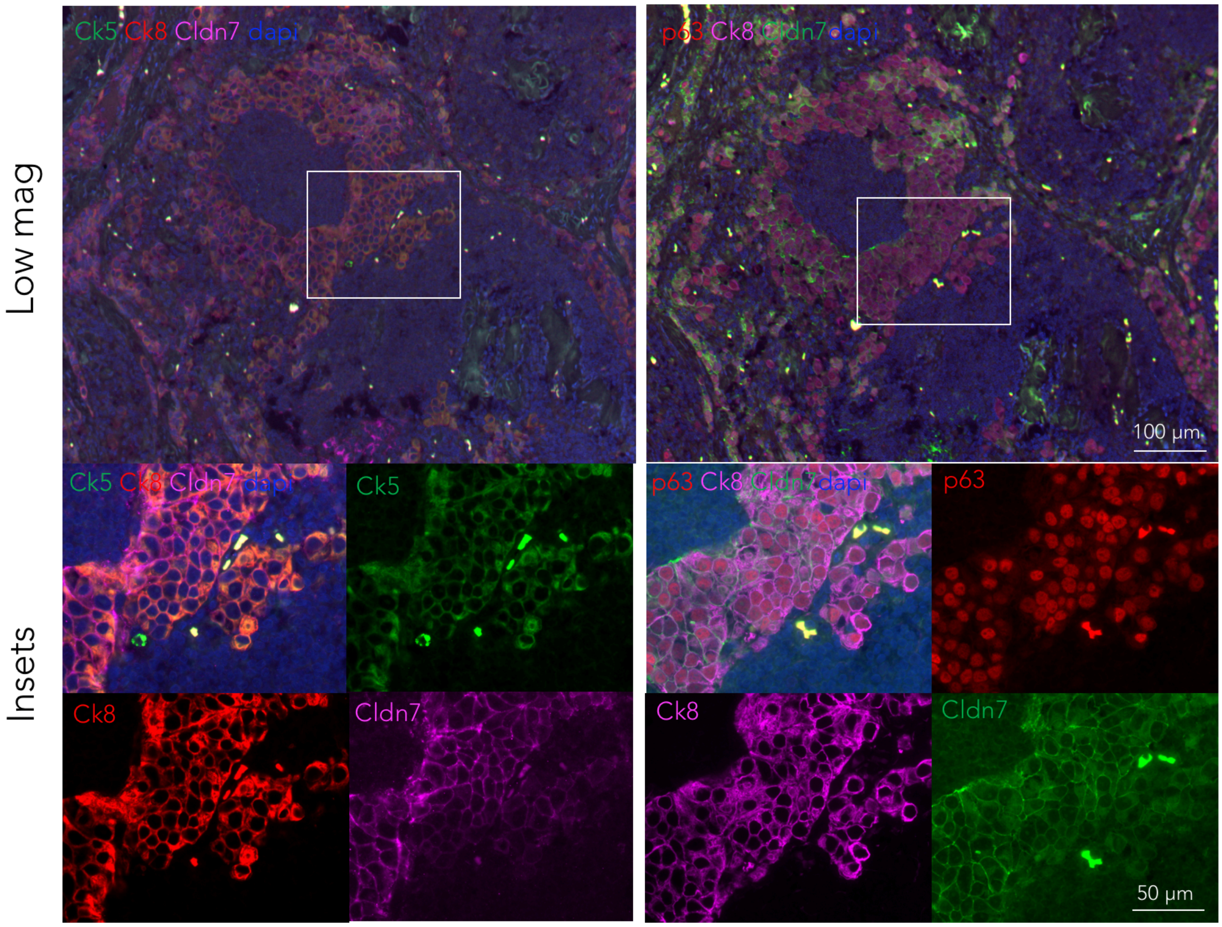

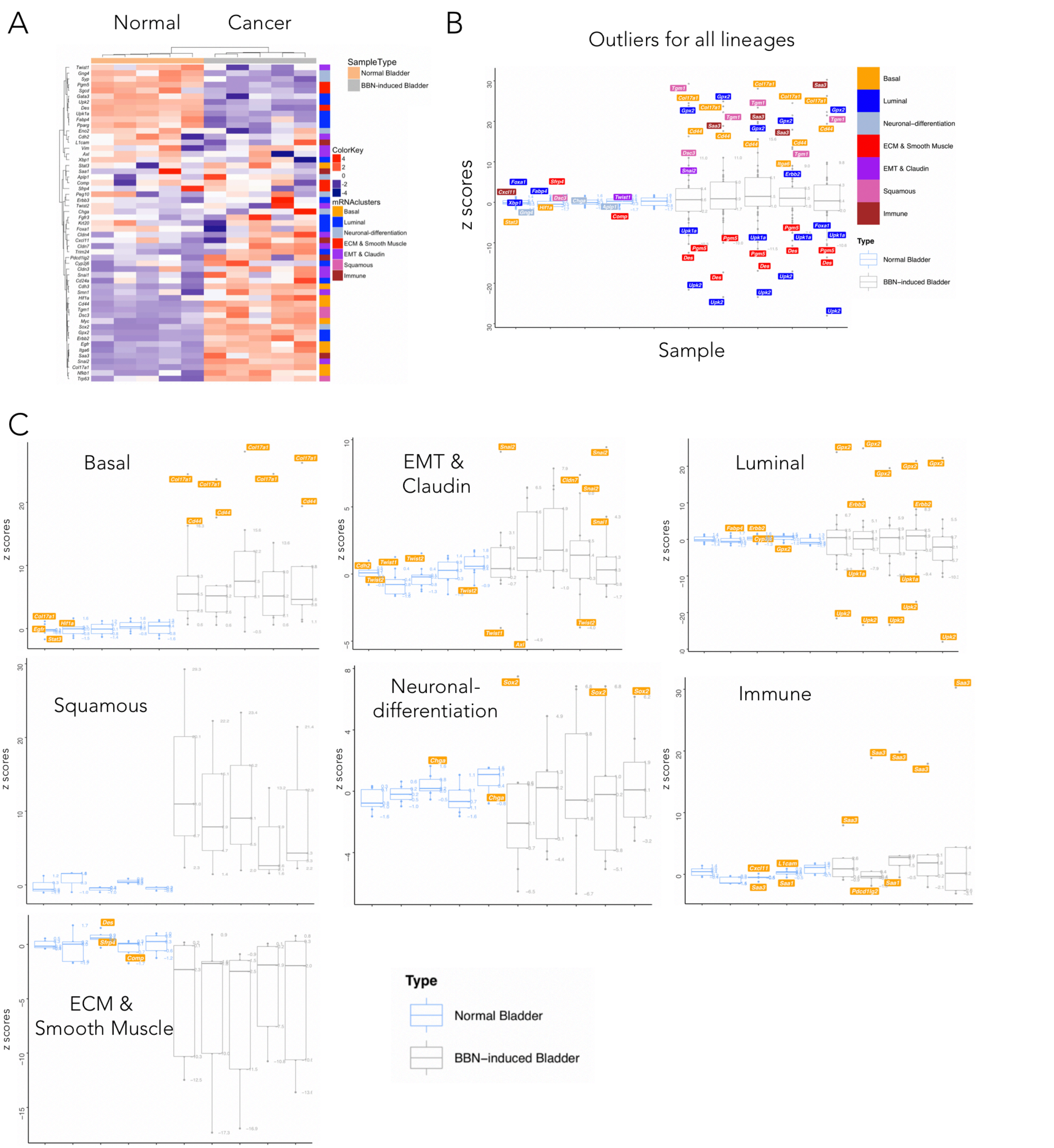

Supplementary Figure 11. Lineage subtyping of OHBBN induced bladder tumors using bulk RNA sequencing analysis. **A,** Heat map showing normal (n=5) and OHBBN treated (n=5) bladders clustered according to mRNA subtypes including basal, luminal, neuronal-differentiated, ECM-smooth muscle, mesenchymal-claudin, squamous and immune. **B,** Box plots comparing z scores of genes for all lineages between normal and OHBBN induced cancer separated by mRNA subtype. Genes shown above and below box plots represent outlier genes for each tumor sample. **C,** Box plots with z scores for genes separated by individual lineages. Clustering analysis were calculated in R and show AIU values = 100, where AU > 95% are statistically significant**.** Box plots showing z scores for gene expression of lineage markers in normal bladders (left, grey boxes) and OHBBN treated (right, grey boxes). Minima, maxima, centre, bounds of box, whiskers are shown.

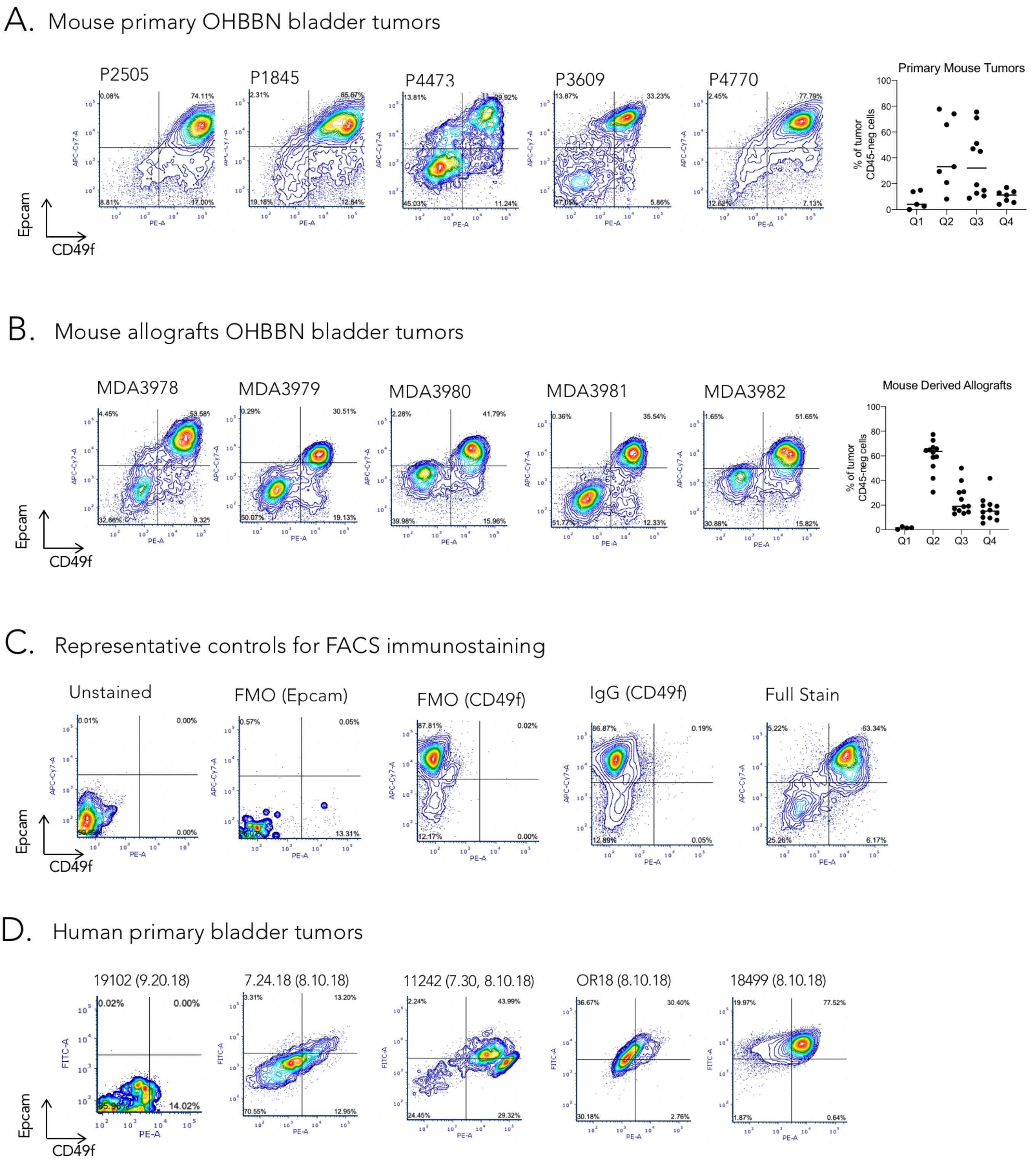

Supplementary Figure 12. Representative flow cytometry analysis showing Epcam and CD49f expression in mouse and human bladder tumors. **A**, primary tumors and scatter plots showing the distribution of Epcam-CD49f expression in quadrants 1-4 (Q1, Q2, Q3, Q4) (n=10). **B**, subcutaneous (MDA) transplanted tumors (n=15) and scatter plots. **C**, representative controls for flow cytometry immunostaining in mouse tumors. **D,** Epcam and CD49f expression in human primary MIBCs (n=17).

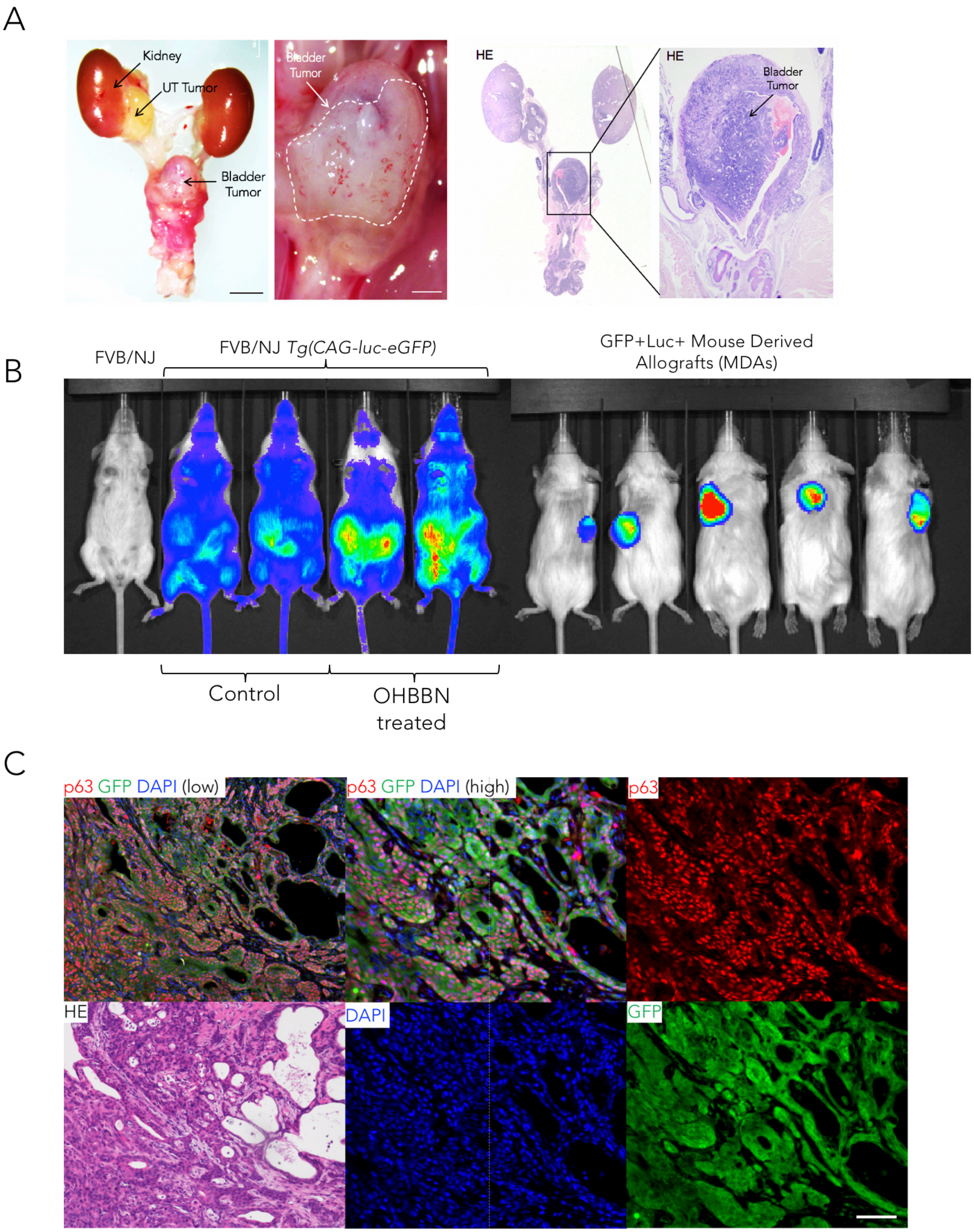

Supplementary Figure 13. Derivation of OHBBN induced bladder tumors. **A,** *Tg(CAG-luc-eGFP)* mice were treated with OHBBN to generate primary bladder tumors as shown at the gross and histological levels (bar = 500 μM) (UT = upper tract). **B, left.** Bioluminescence images of control FVB/NJ mice and *Tg(CAG-luc-eGFP)* FVB/NJ mice treated with OHBBN showing increased bioluminescence in the GU region. **B, right.** Subcutaneous tumors implanted in FVB/NJ mice visualized by bioluminescence. At least 50 independent bladder tumors were assessed. **C,** Co-expression of transgenic GFP and p63 in a *Tg(CAG-luc-eGFP)* FVB/NJ mouse treated with OHBBN (bar = 100 μM). At least 20 independent bladder tumors were assessed.

A

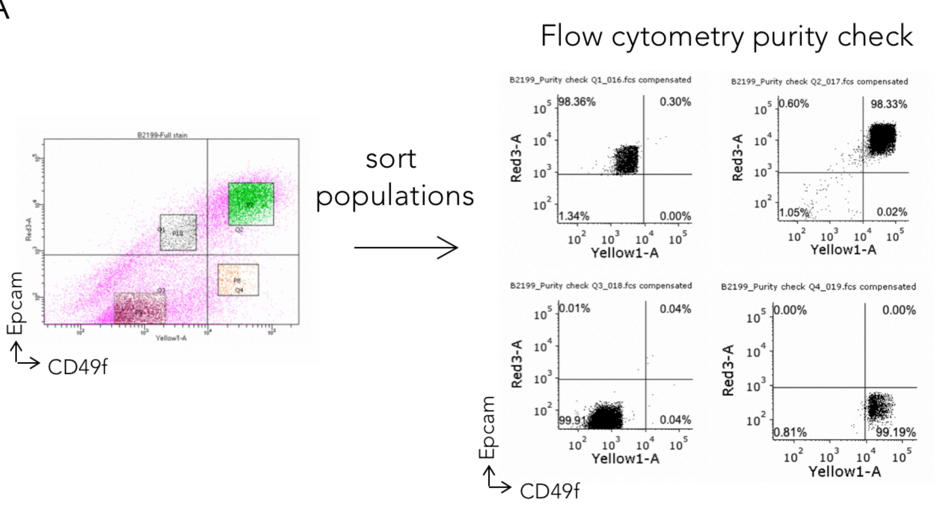

B

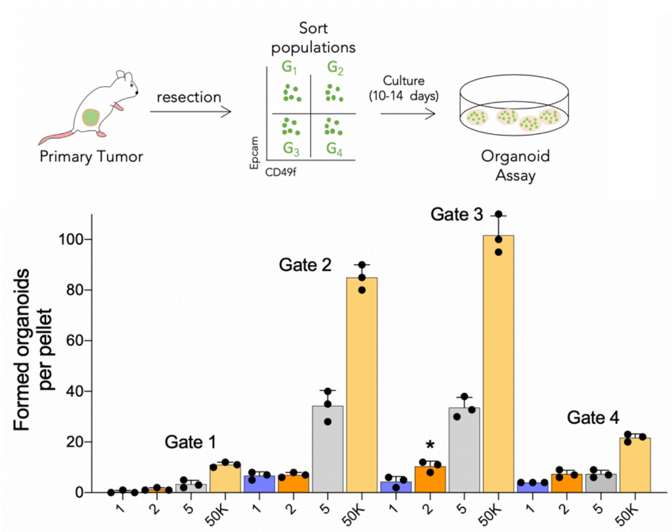

C

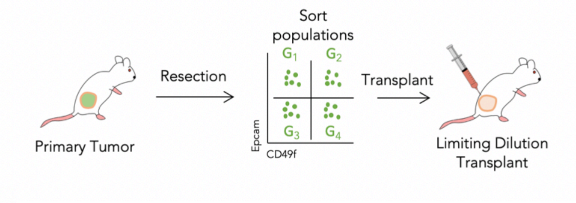

Implant	2K	10K	50K
G1	NG	2/12 (16.6%)	1/6 (16.6%)
G2	NG*	2/10 (20%)	7/8 (87.5%)
G3	NG*	4/10 (40%)	9/10 (90%)
G4	NG	2/ (12.5%)	1/8 (12.5%)

Supplementary Figure 14. Limiting dilutions for *in vitro* and *in vivo* transplantations of sorted tumor cells. A, Representative purity checks for flow cytometry sorted cells. **B,** Comparative *in vitro* evaluation of organoid formation of lineages derived from OHBBN induced primary bladder tumor cells. Cell populations from primary tumors (n=5) were sorted based on CD49f and Epcam expression and plated at variable densities (1, 2 5, or 50,000 cells per 20 µl Cultrex pellet) followed by counting of organoids 10-14 days after plating. Average counts per pellet are shown in bar graphs corresponding to the respective plated cell population. **C,** Comparative evaluation of *in vivo* transplant formation of lineage subpopulations derived from OHBBN induced bladder tumors (primary or P0 tumor). Cell populations based on CD49f and Epcam expression were sorted and injected directly to the primary bladder of recipient FVB mice and evaluated to 8-12 weeks later for formed tumor mass. The efficiencies of tumor formation are shown in absolute and percentage values. NG = no graft formation, NG* = no tumor formation but viable cells present. p values were generated by Students t-test. Individual data points are represented by circles, the error bars represent mean ± standard deviation of the three independent replicates. Each experiment was performed at least four times.
