# Supplementary material for: Epithelial plasticity can generate multi-lineage phenotypes in human and murine bladder cancers
Source: Nat Commun. 2020 May 21;11:2540. doi: 10.1038/s41467-020-16162-3 (PMC7242345; doi:10.1038/s41467-020-16162-3)
Supplement: Supplementary file 3 — Description of Additional Supplementary Files [file 41467_2020_16162_MOESM3_ESM.pdf]

Description of Additional Supplementary Files

File Name: Supplemental Data 1
Description: Top up and down genes from scRNA seq. data for mouse bladder tumors.

File Name: Supplemental Data 2
Description: Top up and down genes from scRNA seq. data for human bladder tumor 357.

File Name: Supplemental Data 3
Description: Top up and down genes from scRNA seq. data for human bladder tumor 359.
