# Supplementary material for: Epithelial plasticity can generate multi-lineage phenotypes in human and murine bladder cancers
Source: Nat Commun. 2020 May 21;11:2540. doi: 10.1038/s41467-020-16162-3 (PMC7242345; doi:10.1038/s41467-020-16162-3)
Supplement: Supplementary file 7 — Reporting Summary [file 41467_2020_16162_MOESM7_ESM.pdf]

natureresearch

Corresponding author(s): David Mulholland
david.mulholland@mssm.edu

Last updated by author(s): 2020.3.21

# Reporting Summary

Nature Research wishes to improve the reproducibility of the work that we publish. This form provides structure for consistency and transparency in reporting. For further information on Nature Research policies, see Authors & Referees and the Editorial Policy Checklist .

## Statistics

For all statistical analyses, confirm that the following items are present in the figure legend, table legend, main text, or Methods section.

| n/a | Confirmed | |
|---|---|---|
| ☐ | ☒ | The exact sample size (*n*) for each experimental group/condition, given as a discrete number and unit of measurement |
| ☐ | ☒ | A statement on whether measurements were taken from distinct samples or whether the same sample was measured repeatedly |
| ☐ | ☒ | The statistical test(s) used AND whether they are one- or two-sided<br>*Only common tests should be described solely by name; describe more complex techniques in the Methods section.* |
| ☐ | ☒ | A description of all covariates tested |
| ☐ | ☒ | A description of any assumptions or corrections, such as tests of normality and adjustment for multiple comparisons |
| ☒ | ☐ | A full description of the statistical parameters including central tendency (e.g. means) or other basic estimates (e.g. regression coefficient) AND variation (e.g. standard deviation) or associated estimates of uncertainty (e.g. confidence intervals) |
| ☐ | ☒ | For null hypothesis testing, the test statistic (e.g. *F*, *t*, *r*) with confidence intervals, effect sizes, degrees of freedom and *P* value noted<br>*Give P values as exact values whenever suitable.* |
| ☒ | ☐ | For Bayesian analysis, information on the choice of priors and Markov chain Monte Carlo settings |
| ☒ | ☐ | For hierarchical and complex designs, identification of the appropriate level for tests and full reporting of outcomes |
| ☒ | ☐ | Estimates of effect sizes (e.g. Cohen's *d*, Pearson's *r*), indicating how they were calculated |

*Our web collection on statistics for biologists contains articles on many of the points above.*

## Software and code

Policy information about availability of computer code

| | |
|---|---|
| Data collection | Single cell RNA sequencing data was collected using Cell Ranger 2.1.1 software |
| Data analysis | Initial post-processing of raw data was performed using Cell Ranger (2.1.1) from 10x genomics. Subsequent data analysis was performed using custom code written in R and is publicly available at: https://github.com/nyuhuyang/scRNAseq-BladderCancer. The following R packages were used: scater, scran, Seurat v3.0, SingleR. For flow cytometry analysis BD FACS Diva 8.0.2 was used. |

For manuscripts utilizing custom algorithms or software that are central to the research but not yet described in published literature, software must be made available to editors/reviewers. We strongly encourage code deposition in a community repository (e.g. GitHub). See the Nature Research guidelines for submitting code & software for further information.

## Data

Policy information about availability of data

All manuscripts must include a data availability statement. This statement should provide the following information, where applicable:

- Accession codes, unique identifiers, or web links for publicly available datasets
- A list of figures that have associated raw data
- A description of any restrictions on data availability

The Human bladder data referenced during the study are available in a public repository from the https://weillcornell.shinyapps.io/Human_BladderCancer/. The mouse bladder data referenced during the study are available in a public repository from https://weillcornell.shinyapps.io/Mouse_BladderCancer/. The differential analysis results are provided as a Source Data file at https://github.com/nyuhuyang/scRNAseq-BladderCancer/tree/master/DEGs. All the other data supporting the findings of this study are available within the article and its supplementary information files and from the corresponding author upon reasonable request. A reporting summary for this article is available as a supplementary information file.

# Field-specific reporting

Please select the one below that is the best fit for your research. If you are not sure, read the appropriate sections before making your selection.

- [x] Life sciences
- [ ] Behavioural & social sciences
- [ ] Ecological, evolutionary & environmental sciences

For a reference copy of the document with all sections, see nature.com/documents/nr-reporting-summary-flat.pdf

# Life sciences study design

All studies must disclose on these points even when the disclosure is negative.

| | |
|---|---|
| Sample size | Sample sizes for in vitro studies included at least 3-6 replicates per assay in order to generate p values by Student's t-test. All in vitro studies were conducted at least 5 times in order to show reproducibility. In vivo studies including transplants and GEM model experiments were reproduced with a sample size of at least 5-10 taking into account any premature animal death and to generate values needed to confirm biological differences in experimental cohorts (p values, Student t test). |
| Data exclusions | No data was excluded from this study. |
| Replication | To verify reproducibility our study used at minimum n=3 for mouse and human bioinformatic analysis. For functional in vitro and in vivo transplantation studies, we conducted methodological replicates (> n=3-5 for each mouse tumor) and at least 3 independent tumors. Experimental observations were reproducible and successful between the independent replicates. |
| Randomization | Mouse IDs associated with carcinogen induced bladder tumors were randomly selected using the RAND function on excel. |
| Blinding | Blinding was not relevant to our study as group allocation was predetermined by the presence of carcinogen or genetically induced cancer or not. |

# Reporting for specific materials, systems and methods

We require information from authors about some types of materials, experimental systems and methods used in many studies. Here, indicate whether each material, system or method listed is relevant to your study. If you are not sure if a list item applies to your research, read the appropriate section before selecting a response.

## Materials & experimental systems

| n/a | Involved in the study | |
|---|---|---|
| ☐ | ☒ | Antibodies |
| ☒ | ☐ | Eukaryotic cell lines |
| ☒ | ☐ | Palaeontology |
| ☐ | ☒ | Animals and other organisms |
| ☐ | ☒ | Human research participants |
| ☒ | ☐ | Clinical data |

## Methods

| n/a | Involved in the study | |
|---|---|---|
| ☒ | ☐ | ChIP-seq |
| ☐ | ☒ | Flow cytometry |
| ☒ | ☐ | MRI-based neuroimaging |

## Antibodies

| | |
|---|---|
| Antibodies used | Flow Cytometry: CD45 (Ms, Biolegend, 103138), CD24 (Ms, Biolegend, 101822), CD44 (Ms/H, Biolegend, 103018), CD49f (Ms/H, 313611), CD49f (Ms/H, 313610), Epcam (Ms, 118208), Epcam (Ms, 118218), Epcam (Ms, 118216), 7AAD (420403). Immunohistochemistry: CK5 (Biolegend, 905501), CK8 (Biolegend, 904801), Epcam (Abcam, ab71916), GATA3 (Abcam, ab106625), Vimentin (Abcam, MA1-37027), Smooth Muscle Actin (MA1-37027), Ki67 (Thermoscientific, RM-9106)<br><br>See Supplementary Figure 17 for antibody dilutions. |
| Validation | Antibody dilutions and means of staining have been included in Supplementary Figure 17. Validations for all antibodies included Isotype (IgG) and FMO staining controls using manufactures suggested antibody dilution. |

## Animals and other organisms

Policy information about studies involving animals; ARRIVE guidelines recommended for reporting animal research

| | |
|---|---|
| Laboratory animals | Our study used FVB/NJ mice treated with OHBBN carcinogen (age 4-6 weeks) to produce mice with bladder cancer (ages 18-24 weeks). Our study also used genetically modified (GEM) mice on a mixed genetic background for lineage tracing analysis (CK5-Tdt) treated with OHBBN at 4-6 weeks and assessed at 18-2 weeks. |

| | |
|---|---|
| | All mice were housed in an AAALAC approved vivarium including hepa filtration, ad libitum supply of food and water, and daily health monitoring. |
| Wild animals | No wild animals were used in this study |
| Field-collected samples | Study did not use field collected animals. |
| Ethics oversight | Use of animals was approved by IACUC protocol (LA13-00060) |

Note that full information on the approval of the study protocol must also be provided in the manuscript.

# Human research participants

Policy information about studies involving human research participants

| | |
|---|---|
| Population characteristics | These two samples are one Male, age 55 (357) and one Female, age 86 (359) with muscle invasive bladder carcinoma samples. Initial presentation of gross hematuria for both. Samples obtained during trans-urethral resection of bladder tumor (TURBT) for diagnosis. Both are treatment naïve samples. 357 was treated with neoadjuvant chemotherapy followed by radical cystoprostatectomy. 359 was treated with chemotherapy and radiation. |
| Recruitment | At our institutions, under approved GU biorepository protocol, all patients with urological cancers, all stages, are consented. This allows for a prospective collection of blood, urine and tissue for experimental use. Samples used in this study were consecutive invasive bladder samples. |
| Ethics oversight | Sample collection and processing was under approval of IRB-10-1180 |

Note that full information on the approval of the study protocol must also be provided in the manuscript.

# Flow Cytometry

## Plots

Confirm that:

- [x] The axis labels state the marker and fluorochrome used (e.g. CD4-FITC).
- [x] The axis scales are clearly visible. Include numbers along axes only for bottom left plot of group (a 'group' is an analysis of identical markers).
- [x] All plots are contour plots with outliers or pseudocolor plots.
- [x] A numerical value for number of cells or percentage (with statistics) is provided.

## Methodology

| | |
|---|---|
| Sample preparation | Flow cytometry was conducted on OHBBN induced mouse and human bladder tumors. For both, tissues were mechanically minced with a razor blade followed by enzymatic digestion using collagenase, rotating for 2-4 hours at 37C. During this time, digestion mixtures were passed 2x through a 21.5G needle and syringe. After digestion, the mixture was passed through a 100M filter and washed in PBS using 2x min cycles of centrifugation at 900 rpm. Pellets were then resuspended in FACS staining buffer (PBS, 1% BSA, 0.05% P/S) to produce single cell mixtures ready for primary antibody staining. |
| Instrument | BD LSR Fortessa Cell Analyzer |
| Software | BD Diva 8.0.2 |
| Cell population abundance | Populations of interest obtained from flow cytometry sorting experiments were shown to have a purity of >98%. This was determined by analyzing a portion of the collected sample and evaluating the % of cells found to be within the original gated population of interest. |
| Gating strategy | For all mouse bladder tumors, single cell isolates were gated on (1) total cells (SSC-A, FCS-A), (2) singlets 1 (FSC-W, FSC-A), (3) singlets 2 (SSC-W, SSC-A), (4) 7AAD-negative, (5) tumor cells + immune cells (GFP, CD45), (6) lineage markers (Epcam, CD49f). Boundaries for positive and negative populations were determined by using FMO (florescence minus one) and non immune IgG isotype controls. Expression above these controls was considered to be positive. |

- [x] Tick this box to confirm that a figure exemplifying the gating strategy is provided in the Supplementary Information.
